# Preparation of Non-Isocyanate Polyurethanes from Mixed Cyclic-Carbonated Compounds: Soybean Oil and CO_2_-Based Poly(ether carbonate)

**DOI:** 10.3390/polym16081171

**Published:** 2024-04-21

**Authors:** Ga Ram Lee, Eun Jong Lee, Hye Sun Shin, Joonwoo Kim, Il Kim, Sung Chul Hong

**Affiliations:** 1 HMC, Department of Nanotechnology and Advanced Materials Engineering, Sejong University, 209 Neungdong-ro, Seoul 05006, Republic of Korea; bennygood2@daum.net (G.R.L.); eunjong088@gmail.com (E.J.L.); 2 Industrial Gas Research TF Team, Particulate Matter Research Center, Research Institute of Industrial Science & Technology (RIST), 187-12 Geumho-ro, Gwangyang-si 57801, Republic of Korea; hyesunshin@rist.re.kr (H.S.S.); realjoon@rist.re.kr (J.K.); 3 Department of Polymer Science and Engineering, Pusan National University, Busan 46241, Republic of Korea; ilkim@pusan.ac.kr

**Keywords:** non-isocyanate polyurethane, cyclic carbonate, poly(ether carbonate), soybean oil, carbon dioxide

## Abstract

This study presents the synthesis and characterization of non-isocyanate polyurethanes (NIPU) derived from the copolymerization of cyclic-carbonated soybean oil (CSBO) and cyclic carbonate (CC)-terminated poly(ether carbonate) (RCC). Using a double-metal cyanide catalyst, poly(ether carbonate) polyol was first synthesized through the copolymerization of carbon dioxide and propylene oxide. The terminal hydroxyl group was then subjected to a substitution reaction with a five-membered CC group using glycerol-1,2-carbonate and oxalyl chloride, yielding RCC. Attempts to prepare NIPU solely using RCC and diamine were unsuccessful, possibly due to the low CC functionality and the aminolysis of RCC’s linear carbonate repeating units. However, when combined with CSBO, solid NIPUs were successfully obtained, exhibiting good thermal stability along with enhanced mechanical properties compared to conventional CSBO-based NIPU formulations. Overall, this study underscores the potential of leveraging renewable resources and carbon capture technologies to develop sustainable NIPUs with tailored properties, thereby expanding their range of applications.

## 1. Introduction

Polyurethane (PU) possesses remarkable versatility in both properties and forms, which can be readily manipulated by adjusting the composition and types of its starting materials [1,2,3,4]. This diverse nature translates to numerous applications across various fields, including automotive, construction, and pharmaceuticals, where PUs appear in various forms like foams, coatings, adhesives, and elastomers [5,6,7]. Conventionally, PUs are synthesized through polyaddition polymerization of isocyanates and polyols, often aided by catalysts and additives [1]. However, the use of isocyanates, derived from hazardous materials like phosgene, raises environmental and health concerns due to their inherent toxicity and moisture sensitivity [8,9,10]. This has led to stricter regulations governing their handling and transport, as emphasized by the REACH (Registration, Evaluation, Authorization, and Restriction of Chemicals) regulation [11,12].

Driven by sustainability and green chemistry principles, recent research in both the academic and industrial spheres has actively explored safer alternatives to isocyanate-based PU production [12,13,14]. These alternatives, termed non-isocyanate polyurethanes (NIPUs), circumvent the use of hazardous isocyanates [8,9,15]. Four primary synthesis routes exist for NIPUs: polycondensation, rearrangement, ring-opening polymerization, and polyaddition [16]. Among these, the aminolysis of cyclic carbonates (CCs), a polyaddition process, yields polyhydroxyurethanes, attracts significant interest due to their industrial and environmental benefits [17,18,19,20]. Compared to isocyanates, CCs offer distinct advantages, such as non-toxicity and moisture insensitivity, leading to safer manufacturing and longer storage times [8,9,16,21]. Notably, NIPU-based coatings are gaining increasing attention due to their ease of handling, low VOC emissions, and superior adhesion and chemical resistance compared to conventional PUs [9,16,21,22,23,24].

Incorporation of CO_2_ into epoxides using renewable bio-based starting materials yields CCs with demonstrably high sustainability credentials [18,25,26,27,28]. Cyclic-carbonated soybean oil (CSBO) exemplifies such materials [9,19,21,27,28]. However, CSBO-derived NIPUs exhibit consistently low tensile strength, averaging around 2~3 MPa (Table S1 and Figure S1, see Supplementary Materials) [29]. This limitation, likely attributed to the flexible aliphatic chains of CSBO, necessitates further improvement [19,22,30,31,32].

**Scheme 1:**
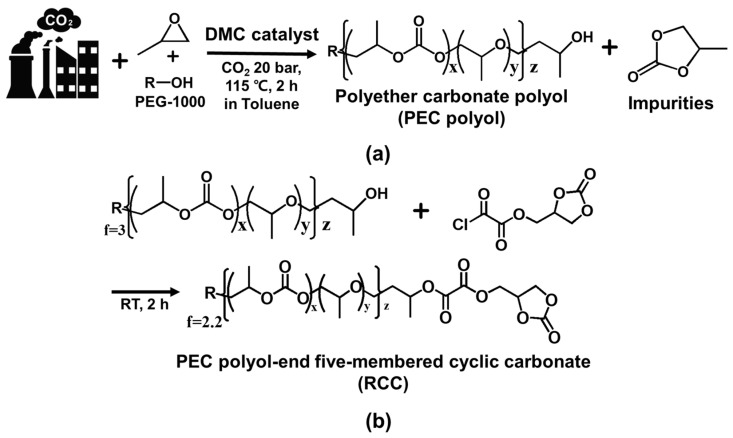
Schematic representation of the preparation of PEC polyol using CO_2_ as a feedstock (**a**) and the preparation of RCC through the end-group substitution reaction of PEC polyol with CC precursor (**b**).

**Scheme 2:**
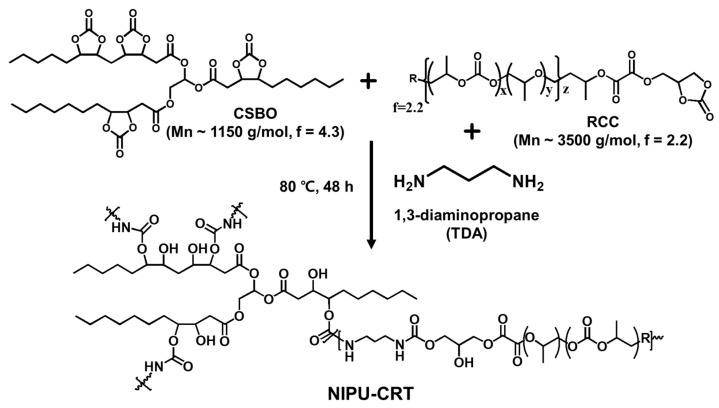
Schematic representation of the preparation of CRT with mixed CC compounds and TDA.

This study aims to address the limitation of low tensile strength in NIPUs derived from CSBO by incorporating CO_2_-based polyether carbonate (PEC) polyol. The rigid carbonate units in CO_2_-based PEC polyol promote strong entanglement between polymer chains, potentially leading to improved tensile strength [33,34,35,36]. Compared to previous similar attempts that employed rigid polyamide components [37], PEC polyol in this study offers several advantages over petroleum-based alternatives. Life cycle assessment studies of the PEC polyol demonstrate reductions in global warming potential (11~19%) and fossil fuel depletion impacts (13~16%) [38]. This translates to an environmentally friendly and industrially relevant NIPU with enhanced mechanical properties. We achieve this by employing a multi-step synthesis approach (Scheme 1). Firstly, PEC polyol is prepared via copolymerization of CO_2_ and epoxide using a double metal cyanide (DMC) catalyst (Scheme 1a) [39,40]. Secondly, five-membered CC-terminated poly(ether carbonate) (RCC) is synthesized by substituting the terminal hydroxyl group of PEC polyol with a CC unit (Scheme 1b). This RCC is then mixed with CSBO and cured with 1,3-diaminopropane (TDA) to form the NIPU (Scheme 2).

## 2. Experimental Section

### 2.1. Materials

Zinc chloride (ZnCl_2_, 99%), 2-ethoxyethyl acetate (EEA, 99%), propylene oxide (PO, 99%), chloroform (99.8%), dichloromethane (>99.9%), acetone (99.5%), toluene (99.5%), ethyl acetate (99.5%), hydrogen peroxide (30.0–35.5%), acetic acid (99.5%), Amberlite IR-120H, tetrabutylammonium bromide (99.0%), and magnesium sulfate (99.5%) were purchased from Samchun Chemicals (Pyeongtaek, Republic of Korea). Potassium hexacyanocobaltate (III) {K_3_[Co(CN)_6_]}, oxalyl chloride (OC, 99%), hexyl amine (HA, 99%), TDA (>99%), sodium bicarbonate (NaHCO_3_, >99.7%), chloroform-d, and poly(ethylene glycol)-*block*-poly(propylene glycol)-*block*-poly(ethylene glycol) (P123, M_n_~5800 g/mol) were purchased from Sigma Aldrich (St. Louis, MO, USA). Dimethyl sulfoxide-d6 (DMSO-d6) was purchased from Eurisotop (Gif-sur-Yvette, France). Glycerol-1,2-carbonate (GC, >90%), propylene carbonate (PC, >98%) were purchased from TCI (Tokyo, Japan). Soybean oil was purchased from Hive company (Wonjoo, Republic of Korea). L(+)-ascorbic acid was purchased from Junsei Chemical (Tokyo, Japan). Glycerol propoxylate (PPG-1000, M_n_ = 1000 g/mol, OH functionality per molecule = 3, Kumho Petrochemical, Seoul, Republic of Korea), CO_2_ (99.999%, Korea Gas Technology, Sangju, Republic of Korea), and all other chemicals were used as received.

### 2.2. Preparation of DMC Catalyst [39]

The DMC catalyst was synthesized using ZnCl_2_ (36 g, 0.26 mol) as the metal salt and K_3_[Co(CN)_6_] (3.9 g, 12 mmol) as the metal cyanide salt with the help of complexing agents. EEA (45 g, 0.34 mol) served as the primary complexing agent, while P123 served as the co-complexing agent. In the preparation process, the solution was initially prepared by dissolving ZnCl_2_ and EEA in 138 mL of water, followed by stirring at 50 °C for 10 min. Separately, another solution, comprising K_3_[Co(CN)_6_] and 16 mL of water, was formulated and then combined with the previous solution, followed by stirring for 1 h. Subsequently, a mixture of EEA (40.5 g, 0.31 mol) and P123 (2.91 g, 0.5 mmol) was added to the solution, followed by stirring for an additional 5 min. Following centrifugation of the solution, washing was performed using two mixtures: a mixture of EEA (40.5 g, 0.31 mol)/distilled water (60 mL) and a mixture of EEA (40.5 g, 0.31 mol)/P123 (1.5 g, 0.3 mmol). The resulting precipitate was collected and washed repeatedly with distilled water and EEA. Finally, the DMC catalyst was obtained by vacuum drying at 80 °C overnight.

### 2.3. Preparation of PEC Polyol [40]

A high-pressure reactor (Model No. 4568, Parr Instrument Company, Moline, IL, USA) was utilized for the copolymerization of PO and CO_2_ in the presence of the synthesized DMC catalyst. For the synthesis of PEC polyol, PPG-1000 served as the initiator. The reactor was charged with 0.11 g of the DMC catalyst and 90 mL of toluene, followed by introducing CO_2_ to pressurize the reactor to 20 bar at 115 °C. Then, a mixture of PO (74.7 g, 90 mL) and PPG-1000 (20 mL) was fed into the reactor using an HPLC pump over a period of 90 min, initiating the polymerization. The reaction continued for an additional 30 min before being terminated by cooling the reactor to room temperature and venting the CO_2_. The product mixture was first concentrated by evaporation to remove the solvent and unreacted PO monomer. Subsequently, the concentrated mixture was filtered and washed with chloroform to remove the DMC catalyst. Finally, the remaining mixture was thoroughly washed with distilled water to remove PC by-products and isolate the desired PEC polyol (61.3 g).

### 2.4. Preparation of Five-Membered CC-Terminated Poly(Ether Carbonate) (RCC)

A five-membered CC precursor was synthesized using GC and OC (Figure S2, see Supplementary Materials). In a 1000 mL three-necked round-bottom flask, OC (138 g, 1.1 mol) was dissolved in 170 mL of dichloromethane under a nitrogen atmosphere. Separately, GC (47 g, 0.4 mol) was dissolved in 200 mL of dichloromethane. The GC solution was then added dropwise to the flask containing OC with vigorous stirring. The reaction proceeded for 2 h at room temperature, followed by the removal of unreacted OC by vacuum in an ice bath. The resulting precursor was dried under vacuum at 80 °C overnight. RCC was then synthesized by reacting the PEC polyol with the CC precursor in a 1:3 molar ratio. The CC precursor was first dissolved in dichloromethane, followed by the addition of the PEC polyol in dichloromethane. The reaction mixture was stirred for 2 h at room temperature, followed by the isolation of the desired product by multiple washings with a 5% aqueous NaHCO_3_ solution and distilled water.

### 2.5. Preparation of CSBO

A two-step process involving epoxidation and subsequent carbonation was employed for CSBO synthesis. Initially, soybean oil (100 g, 0.11 mol) was dried under vacuum at 80 °C overnight. In a three-neck flask equipped with a dropping funnel, the dried oil, acetic acid (15 g, 0.25 mol), Amberlite IRC-120H (25 g), and toluene (18) were combined. The reaction mixture was stirred at 45 °C while hydrogen peroxide (66.1 g, 1.94 mol) was added dropwise over 8 h. After the reaction, the mixture was filtered to remove Amberlite IRC-120H and washed with distilled water until a neutral pH was achieved. Purification was performed using magnesium sulfate, followed by solvent evaporation at 100~110 °C, yielding epoxidized soybean oil. For CSBO synthesis, epoxidized soybean oil (20 g), tetrabutylammonium bromide (112 g, 0.35 mol), and ascorbic acid (22.9 g, 0.13 mol) were charged into a high-pressure reactor. The reactor was purged with CO_2_ gas for 10 min, then pressurized to 34.5 bar under a CO_2_ atmosphere. The reaction mixture was stirred at 80 °C for 14 h. After completion, ethyl acetate (18 mL) was added to extract the product mixture. The extract was washed with distilled water and purified using magnesium sulfate. Finally, ethyl acetate was removed by vacuum evaporation at 70 °C for 2 h and then at 110 °C for 5 h, resulting in CSBO (M_n_~1150 g/mol, CC functionality~4.3).

### 2.6. Model Reaction of CC with Amine

PC (2.00 g, 1.96 × 10^−2^ mol) and HA (1.98 g, 1.96 × 10^−2^ mol) were equimolarly mixed in a 50 mL round-bottom flask under a nitrogen atmosphere. The mixture was stirred at 80 °C for 24 h to induce the formation of a reaction product with a urethane linkage. The progression of the reaction was monitored using Fourier transform infrared (FT-IR) and proton nuclear magnetic resonance (^1^H NMR) spectroscopy. FT-IR: 3622~3138 cm^−1^, O-H stretching and N-H stretching; 1687 cm^−1^, urethane C=O stretching peak; 1533 cm^−1^, N-H bending; and 1249 cm^−1^, C-O stretching. ^1^H NMR (DMSO-d6, 500 MHz), δ (ppm): 6.95~7.14 (d, 1H), 4.70~4.77 (m, 1H), 4.56~4.65 (m, 1H), 3.69~3.85 (m, 2H), 3.30~3.43 (m, 2H), 2.91~2.98 (m, 2H), 1.20~1.42 (m, 9H), 1.02~1.12 (m, 3H), and 0.83~0.89 (t, 3H).

### 2.7. Preparation of NIPUs (RT and CRT)

A schematic representation of how to prepare NIPU with CSBO, RCC, and TDA (CRT) is shown in Scheme 2. RCC (0.2 g) and CSBO (1.8 g) were vigorously mixed in a Teflon-coated iron mold for 1 min at room temperature. Subsequently, TDA was introduced to the mixture and further mixed for 2 min at room temperature. The homogenized mixture was then poured into a Teflon mold conforming to ASTM D-638 [41] type V specifications and cured at 80 °C for 48 h to yield the desired reaction products (CRTs). For NIPU prepared with RCC and TDA without CSBO (RT), the aforementioned procedure was followed, albeit with a reduced reaction time of 24 h. The formulations to prepare RT and CRTs are summarized in Table 1.

### 2.8. Characterization

To investigate the reactivity of CCs towards amines and to characterize the structural attributes of PEC polyol and RCC, ^1^H NMR analyses were performed using a Bruker 500 MHz AvanceIII HD500 spectrometer (Billerica, MA, USA). These analyses were conducted at room temperature, utilizing chloroform-d or DMSO-d6 as solvents. The conversion of PC via ring-opening reactions with HA was assessed by analyzing the area values under the ^1^H NMR signals, following Equation (1).

(1) $$\mathrm{Conversion}\;\mathrm{of}\;\mathrm{PC}\;\left(\%\right)=\frac{A_{7.05}}{A_{4.06}+A_{7.05}}\times 100,\;$$

where A_4.06_ and A_7.05_ denoted the characteristic area values of the ^1^H NMR signal at 4.06 ppm of HA and 7.05 ppm of the product, respectively. The CC functionality of RCC was also assessed using ^1^H NMR spectroscopy, as described by Equation (2).

(2) Cycic carbonate functionality of RCC =Hydroxyl functionality of PEC polyol ×A5.23A3.94,

where A_3.94_ represents the area value of the ^1^H NMR signals at 3.94 ppm, which corresponds to the C-H peak of PEC polyol. Similarly, A_5.23_ denotes the area value of the ^1^H NMR signals at 5.23 ppm, corresponding to the C-H peak of RCC. ^13^C NMR analyses were performed at room temperature using a Bruker 600 MHz AvanceIII 600 spectrometer (Billerica, MA, USA) with chloroform-d as the solvent. The hydroxyl values (mg-KOH/g) of PEC polyol and RCC were determined via volumetric titration according to ASTM D1957-86 [42], utilizing Equation (3) from the standard.

(3) $$\mathrm{Hydroxyl}\;\mathrm{value}=\frac{\left(A-B\right)\times \;N\times 56.1}{S},$$

where A is the volume (mL) of the potassium hydroxide solution used in the blank test, B is the volume (mL) of the potassium hydroxide solution used in the sample titration, N is the normality of the potassium hydroxide solution, and S is the weight (g) of the sample. Gel permeation chromatography (GPC) was conducted using a Shimadzu Nexera system (Kyoto, Japan) equipped with a PSS column (Styragel HR 2, 4, and 5) and a Shimadzu RID-20A refractive index detector (Kyoto, Japan). THF of high-performance liquid chromatography (HPLC) grade served as the eluent, flowing at a rate of 1.0 mL/min. Calibration of the system was executed employing linear polystyrene (PS) standards spanning a molecular weight range of 641 to 13.5 × 10^6^ g/mol. FT-IR spectra were acquired using a Thermo Fisher Scientific Nicolet 380 (Waltham, MA, USA) spectrometer. For CRT samples, analysis was conducted in attenuated total reflection mode with a resolution of 4 cm^−1^ and 64 scans, covering a wavenumber range of 4000 to 650 cm^−1^. Other samples were analyzed in transmission mode. The thermal degradation characteristics of the synthesized NIPU samples were examined through thermogravimetric analysis (TGA) using an SDT-650 instrument from TA Instruments (New Castle, DE, USA). Samples weighing 5 mg were subjected to heating from 30 °C to 700 °C under a nitrogen atmosphere, with a heating rate of 10 °C/min. The gel content of the NIPU samples was determined according to Equation (4). Initially, dry samples (W_0_) were submerged in acetone for 48 h. Subsequently, the solvent was removed and evaporated under vacuum at 70 °C for 12 h to obtain the final weight (W_1_).

(4) $$\mathrm{Gel}\;\mathrm{content}\;\left(\%\right)=\frac{W_{1}}{W_{0}}\times 100\%$$

The mechanical properties of the NIPU samples were assessed using a universal testing machine (AGS-5kNX, Shimadzu) under tensile testing conditions. Following ASTM D-638, standard type V dumbbell-shaped specimens were tested at room temperature with a crosshead speed of 10 mm/min and a gauge length of 7.6 mm.

## 3. Results and Discussion

**Figure 1:**
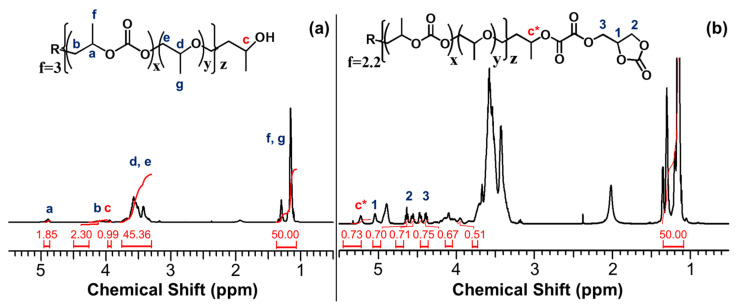
^1^H NMR spectra of PEC polyol (**a**) and RCC (**b**).

PEC polyol was synthesized via the polymerization of CO_2_ with PO, employing a DMC catalyst. The structural features of PEC polyol were elucidated through ^1^H NMR analysis. As depicted in Figure 1a, the signals at 1.1~1.3 ppm corresponded to protons of the CH_3_ group in both the propylene ether and PC units. The signals at 3.3~3.8 ppm corresponded to protons of CH and CH_2_ in the propylene ether unit. Additionally, signals at 4.1~4.2 ppm and 4.9~5.0 ppm corresponded to protons of CH_2_ and CH in the PC unit, respectively. The ^1^H NMR analysis showed that the PEC polyol consisted of 73.7 wt% propylene ether units and 26.3 wt% propylene carbonate units. Peaks at chemical shifts of 3.9~4.0 ppm were characteristic of the CH group adjacent to the terminal hydroxyl group. The ^13^C-NMR spectrum of PEC polyol is provided in Figure S3a (see Supplementary Materials). The mass spectrum of PEC polyol is provided in our previous literature, demonstrating the co-existence of propylene ether and propylene carbonate units in the polymer chain [43].

**Figure 2:**
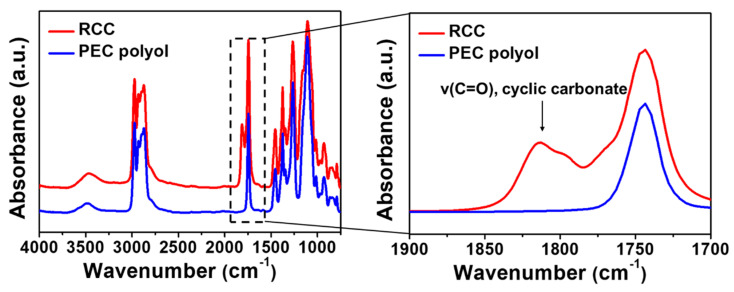
FT-IR spectra of PEC polyol and RCC.

The hydroxyl group of PEC polyol underwent substitution with a CC group using the product from the reaction between GC and OC to yield RCC (Scheme 1b). The chemical structure of RCC was investigated through ^1^H NMR and FT-IR analyses (Figure 1b). Proton signals observed at 4.4~5.1 ppm confirmed the formation of the introduced CC units. Additionally, the peak at 4.1~4.2 ppm of PEC polyol shifted to the c* peak at 5.2~5.3 ppm. The ^13^C-NMR spectrum of RCC (Figure S3b, see Supplementary Materials) also shows the presence of terminal CC units. In contrast to PEC polyol, the presence of a carbonyl peak at 1812 cm^−1^ in the FT-IR spectrum of RCC further confirmed the incorporation of CC units (Figure 2).

The hydroxyl values of PEC polyol and RCC were determined using the titration method following ASTM D1957-86 (Table 2). The values were 54.89 and 12.37 mg-KOH/g for PEC polyol and RCC, respectively. These values translated to hydroxyl chain-end functionalities of 2.94 and 0.77 for PEC polyol and RCC, respectively, calculated from the hydroxyl values and their molecular weights. The substitution of the terminal hydroxyl group in PEC polyol with the CC unit in RCC corresponds to the simultaneous disappearance of a hydroxyl group and the formation of a CC unit. Therefore, the CC functionality of RCC (2.17, Table 2) was calculated by the difference in hydroxyl functionality values between PEC polyol and RCC. This value well agreed with the CC functionality determined using ^1^H NMR (Figure 1) and Equation (2), which also yielded a CC chain-end functionality value of 2.17 for RCC.

**Figure 3:**
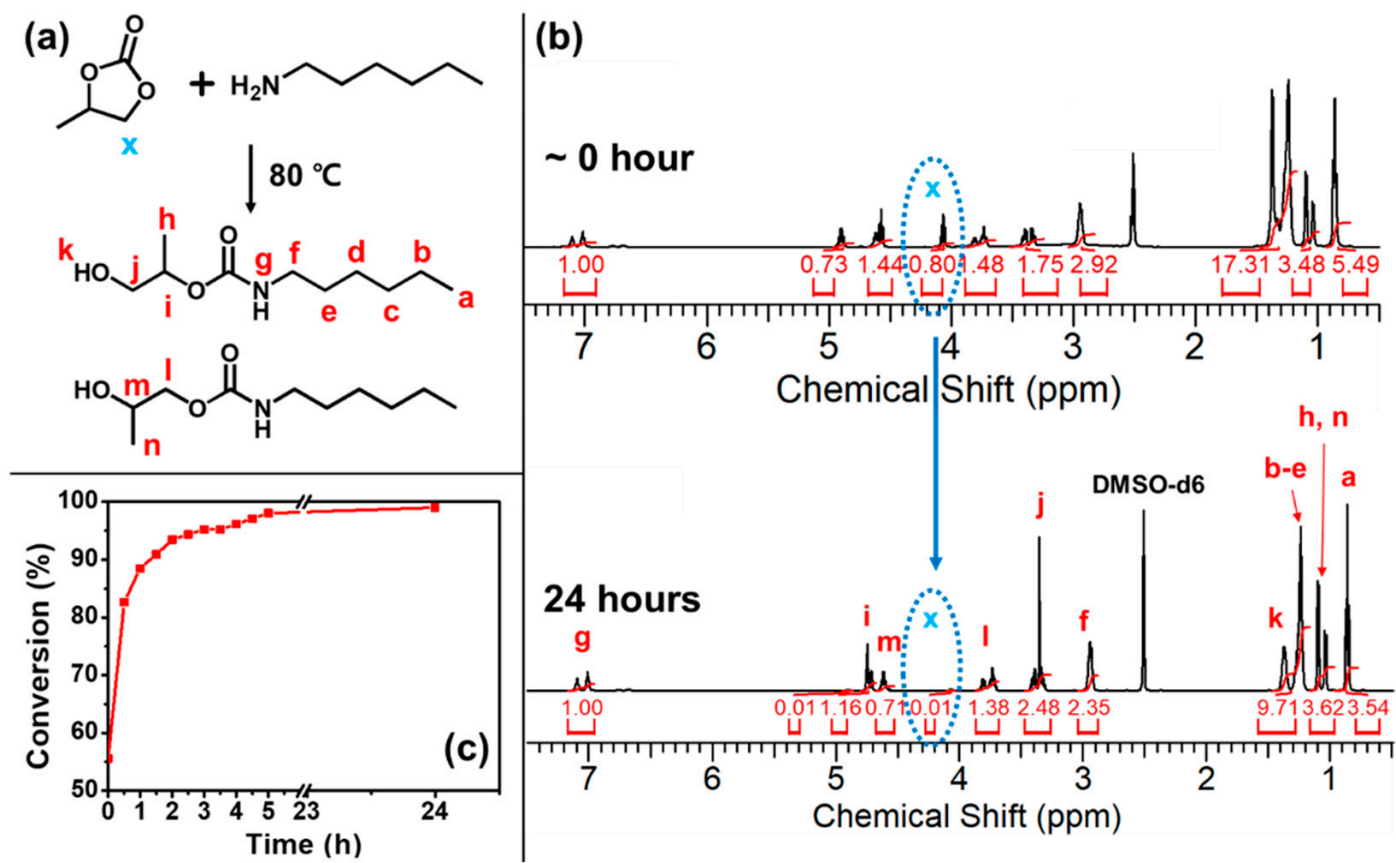
Schematic representation of the preparation of urethane products by the reaction between PC and HA (**a**), ^1^H NMR spectra of the urethane products (**b**), and conversion of CC as a function of reaction time (**c**).

**Figure 4:**
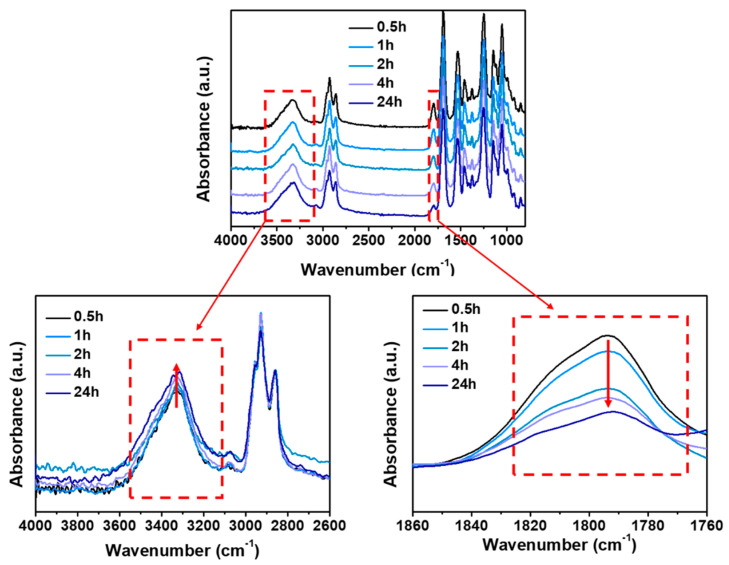
FT-IR spectra of the urethane product prepared by the reaction between PC and HA as a function of time at 80 °C.

To evaluate the reactivity of the CC unit for the ring-opening reaction with amine, a model reaction between PC and HA was initially studied (Figure 3a). The formation of urethane linkages over time was monitored using both FT-IR and ^1^H NMR spectroscopy. In ^1^H NMR spectra, signals between 6.95 and 7.14 ppm were attributed to protons associated with the newly formed urethane bonds (Figure 3b). To optimize reaction conditions, the conversion of CC units to urethane linkages was calculated based on Figure 3b and Equation (1). The results demonstrated that at 80 °C, over 80% conversion was achieved within 30 min, with near-complete conversion observed after 24 h (Figure 3c). Similarly, Figure 4 reveals a gradual decrease in the cyclic carbonyl peak at 1790 cm^−1^ in the FT-IR spectra with increasing reaction time, indicating the consumption of CC units. This coincided with a slight increase in the peak at 3320 cm^−1^, likely attributed to the newly formed O-H groups in the reaction product.

**Figure 5:**
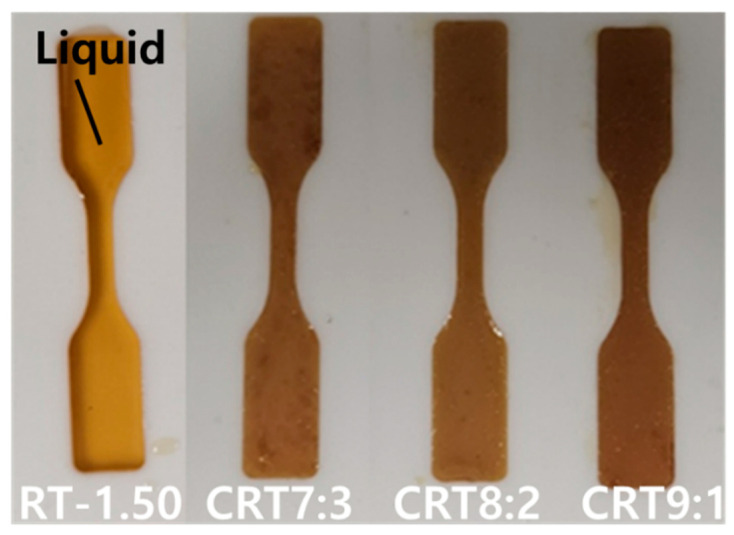
Visual appearances of CRTs in Teflon molds.

The preparation of NIPU using RCC and TDA was then attempted. TDA was employed as a diamine species in this study due to its good miscibility with RCC. The reaction, however, appeared to be unsuccessful, as shown in Figure S4 (see Supplementary Materials), where no solidification of the reaction mixture was observed. In addition to the low CC functionality (f~2.2) of RCC, the linear carbonate repeating units in RCC can also be cleaved through aminolysis reactions in the presence of amines [44,45,46]. Despite attempts to control the amount of the amine to minimize aminolysis of linear carbonate units (RT-0.85~1.50, Table 1), the preparation of solid NIPU did not occur. Even with an increased amine content and an extended reaction time, RT-1.50 still exhibited a liquid state (Figure 5, RT-1.50).

CSBO is a type of soybean oil that has undergone a chemical reaction to incorporate five-membered CC units in its chemical structure. The process typically includes the reaction of epoxidized soybean oil with carbon dioxide, exemplifying “carbon capture and usage” technologies to provide environmentally friendly products. CSBO can further react with diamines to produce NIPU networks [25,37,47]. Utilizing the reaction capability of CC units in CSBO, the preparation of NIPU (CRT) using a mixture of CSBO and RCC was thus attempted (Scheme 2).

**Figure 6:**
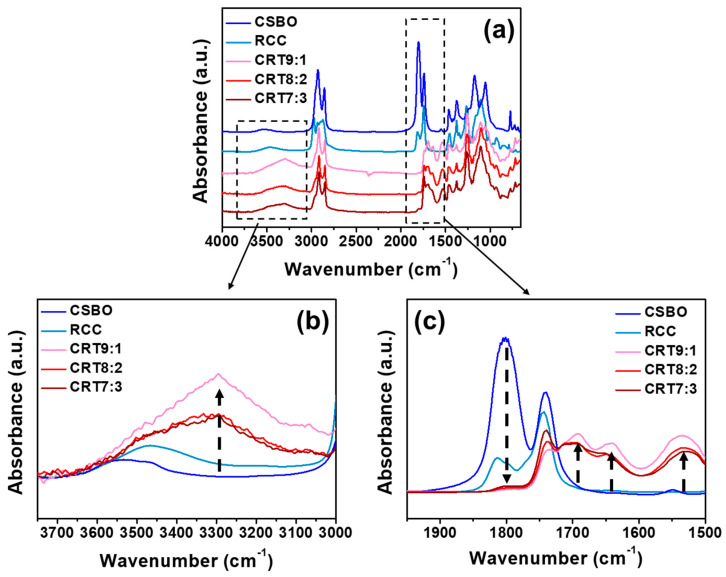
FT-IR spectra of CRTs in the range of 650~4000 cm^−1^ (**a**); enlarged spectra in the range of 3000~3750 cm^−1^ (**b**) and 1500~1950 cm^−1^ (**c**).

The successful synthesis of solidified NIPU, i.e., CRTs, is demonstrated in Figure 5, resulting in brown ASTM D-638 Type V specimens. The successful preparation of CRTs was also evidenced by the appearance of characteristic peaks of urethane linkages in FT-IR spectra (Figure 6). In Figure 6b, the broad absorption peaks in the range of 3100~3600 cm^−1^ were attributed to the O-H and N-H stretching peaks originating from the ring-opening reaction of the CC with amine (Figure 6b) [48,49]. Furthermore, the distinct appearance of the urethane carbonyl stretching peak at 1692 cm^−1^ and the N–H bending peak at 1532 cm^−1^ corresponded to the formation of urethane linkages (Figure 6c). The disappearance of the CC carbonyl peak at 1802 cm^−1^ provided clear support for the ring-opening reaction of CC units in CSBO/RCC mixtures to form NIPU (Figure 6c) [23,50]. Additionally, the absorption peak of amide at 1648 cm^−1^ was barely noticeable, indicating that the aminolysis of ester groups of CSBO was not significant [23,50].

**Figure 7:**
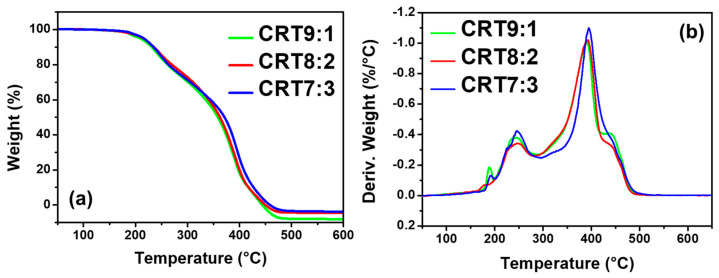
TGA (**a**) and their DTG (**b**) curves of CRTs.

The thermal stability of CRTs prepared with different CSBO/RCC ratios was evaluated using TGA (Figure 7a). The T_10_, T_25_, and T_50_ values correspond to 10%, 25%, and 50% weight loss of CRTs, respectively (Table 3). The T_10_ values, indicating the onset of decomposition, were all higher than the curing temperature of 80 °C, suggesting the formation of molecular network structures within CRTs during the reaction [51,52]. Notably, the decomposition temperature values (T_10_, T_25_, and T_50_) for CRT8:2 and CRT7:3 were overall higher than those of CRT9:1 (Table 3), implying that the CRTs with a higher RCC content exhibit enhanced thermal stability.

The derivative thermogravimetric (DTG) curves of the CRTs (Figure 7b) revealed distinct thermal decomposition stages. The first stage, occurring at 200~300 °C, corresponded to the degradation of urethane linkages within CRTs. The second stage, attributed to the degradation of the fatty acid chains of CSBO, occurred at higher temperatures [53,54]. A final, minor stage above 400 °C was ascribed to the decomposition of carbonate units present in the RCC [43,55].

**Figure 8:**
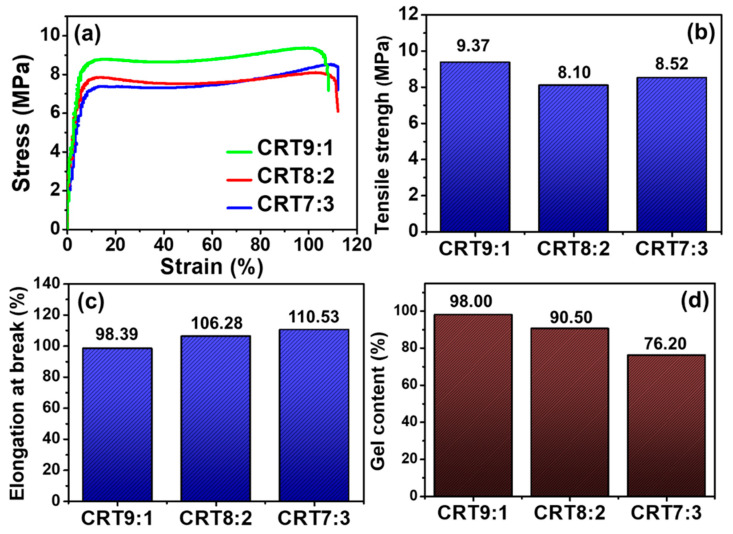
Stress–strain curves (**a**), tensile strength (**b**), elongation at break (**c**), and gel content (**d**) of CRTs.

The tensile mechanical properties of the synthesized CRTs were evaluated using a universal tensile testing machine. Figure 8 illustrates the plastic deformation behaviors of the CRTs, which are characterized by tensile strengths ranging from 8 to 10 MPa and elongation at break values between 100 and 110%. Notably, previous studies focused on NIPU preparation using CSBO and diamine reported lower tensile strengths, typically ranging from 1 to 4 MPa, which obviously was a soft elastomeric behavior (Table S1, see Supplementary Materials) [25,30,56]. In contrast, the CRTs synthesized in this study from CSBO/RCC mixtures exhibited a ductile plastic behavior with considerably higher tensile strengths (8~10 MPa), as presented in Figure S1 (see Supplementary Materials). This enhanced mechanical performance is likely attributed to the incorporation of RCC alongside CSBO in the NIPU structure. The relatively high molecular weight of RCC with stiff carbonate repeating units contributes to the formation of a denser and more rigid polymer network [54,57]. As the CSBO content increased, a trend of increasing tensile strength and decreasing elongation at break was observed for the CRTs (Figure 8b,c). This phenomenon aligns with the gel content analysis (Figure 8d), which revealed a natural increase in cross-linking with higher CSBO content due to its multi-cyclic carbonate functionality [56,58]. This increased cross-linking density contributes to the observed higher tensile strength and lower elongation at break values in the resulting CRTs [56].

## 4. Conclusions

This study presents the synthesis and characterization of NIPU derived from CO_2_, PO, GC, and CSBO. The synthesis of PEC polyol and its subsequent reaction to form CC-terminated RCC were successfully achieved, as confirmed by ^1^H NMR, FT-IR, and hydroxyl value analyses. The reactivity of the CC units was explored through a model reaction of PC and HA, confirming the ability of CC units to form urethane linkages. Attempts to prepare NIPU using RCC and TDA were unsuccessful, possibly due to the low CC functionality and the aminolysis reaction of RCC’s linear carbonate repeating units. However, when combined with CSBO, the resulting CRTs exhibited improved mechanical properties, attributed to the combined effects of RCC and CSBO in forming a cross-linked polymer network. The incorporation of CSBO contributed to reasonable thermal resistance and increased tensile strength, indicating better performance than conventional CSBO-based NIPU formulations. Overall, the successful synthesis of CRTs highlighted the potential of utilizing renewable resources and carbon capture technologies in the development of sustainable polymeric materials with tailored properties. Further research may focus on optimizing the synthesis parameters and exploring additional functionalizations to expand the range of applications for these novel materials.

## Figures and Tables

**Table 1:** Formulations to prepare NIPUs *.

Designation	RCC		CSBO		TDA		[CC]:[NH_2_]	Time (h)
	g	mmol-CC	g	mmol-CC	g	mmol-NH_2_		
RT-0.85	3.000	1.886	-	-	0.059	1.603	1:0.85	24
RT-1.00	3.000	1.886	-	-	0.070	1.886	1:1.00	
RT-1.15	3.000	1.886	-	-	0.080	2.169	1:1.15	
RT-1.30	3.000	1.886	-	-	0.091	2.451	1:1.30	
RT-1.50	2.000	1.257	-	-	0.070	1.886	1:1.50	48
CRT7:3	0.600	0.377	1.400	5.226	0.311	8.404		
CRT8:2	0.400	0.251	1.600	5.972	0.346	9.335		
CRT9:1	0.200	0.126	1.800	6.719	0.380	10.267		

* Reaction temperature = 80 °C.

**Table 2:** Hydroxyl and cyclic carbonate functionalities of PEC polyol and RCC.

	Molecular Weight ^a^ [g/mol]	Hydroxyl Value ^b^ [mgKOH/g]	OH Functionality ^c^	Cyclic Carbonate Functionality ^d^
PEC polyol	3000	54.89	2.94	-
RCC	3500	12.37	0.77	2.17

^a^ Determined by GPC. ^b^ Determined by volumetric titration according to ASTM D1957-86. ^c^ OH functionality = hydroxyl value × molecular weight/56,100. ^d^ Cyclic carbonate functionality = OH functionality of PEC polyol-OH functionality of PECC.

**Table 3:** Thermogravimetric characteristics of CRTs.

Designation	T_10_ (°C)	T_25_ (°C)	T_50_ (°C)
CRT9:1	231	281	359
CRT8:2	235	291	362
CRT7:3	235	283	371

## Data Availability

The data presented in this study are available within this article.

## Supplementary Materials

The following supporting information can be downloaded at: https://www.mdpi.com/article/10.3390/polym16081171/s1, Figure S1: Tensile strength and elongation at break comparison of CSBO-based NIPU: Our work versus previous studies; Figure S2: Schematic representation of the preparation of five-membered CC precursor; Figure S3: 13C NMR spectra of PEC polyol (a) and RCC (b); Figure S4: Visual appearances of RTs in iron mold; Table S1: Examples of CSBO-based NIPU. References [25,30,47,48,51,56,57,59] are cited in the Supplementary Materials.
