# Supplementary material for: Preparation of Non-Isocyanate Polyurethanes from Mixed Cyclic-Carbonated Compounds: Soybean Oil and CO2-Based Poly(ether carbonate)
Source: Polymers (Basel). 2024 Apr 21;16(8):1171. doi: 10.3390/polym16081171 (PMC11053720; doi:10.3390/polym16081171)
Supplement: Supplementary file 1 [file polymers-16-01171-s001.zip › polymers-2938042-supplementary.pdf]

Supplementary Material

Preparation of Non-Isocyanate Polyurethanes from Mixed Cyclic-Carbonated Compounds: Soybean Oil and CO2-Based Poly(ether carbonate)

Table S1. Examples of CSBO-based NIPU

Amine	[CC]:[NH_2]	Tensile strength (MPa)	Elongation at break (%)	Reference
1,2-ethylenediamine	1:0.5	0.49	72	[1]
	1:1	5.77	90	
	1:2	1.75	198	
1,4-butylenediamine	1:0.5	0.84	87	
	1:1	4.71	131	
	1:2	3.28	219	
1,6-hexamethylenediamine	1:0.5	0.74	71	
	1:1	3.80	189	
	1:2	2.67	207	
3-aminopropyl-terminated poly (ethylene glycol)	-	3.17	49	[2]
1,9-nonanediamine	1:1	3.40	132	[3]
1,13-tridecanediamine	1:1	3.20	128	
Diethylene glycol bis(3-aminopropyl) ether	1:0.5	0.85	145	[4]
Priamine™ 1074	1:0.5	0.92	233	
1,8-Menthane diamine	1:0.8	1.45	108	[5]
	1:1	2.20	101	
	1:1.2	1.59	106	
4,4-diaminodiphenyl methane	1:0.8	1.69	117	[6]
	1:1	2.56	142	
	1:1.2	2.02	105	
4,4-diaminodiphenyl disulfide	1:1	1.99	166	[7]
m-Xylylenediamine	1:1	3.3	200	[8]

References of Table S1

1. Javni, I.; Hong, D.P.; Petrović, Z.S. Soy-based polyurethanes by nonisocyanate route. *J. Appl. Polym. Sci.* **2008**, *108*, 3867-3875.

2. Jalilian, S.; Yeganeh, H. Preparation and properties of biodegradable polyurethane networks from carbonated soybean oil. *Polym. Bull.* **2015**, *72*, 1379-1392.

3. Samanta, S.; Selvakumar, S.; Bahr, J.; Wickramaratne, D.S.; Sibi, M.; Chisholm, B.J. Synthesis and characterization of polyurethane networks derived from soybean-oil-based cyclic carbonates and bioderivable diamines. *ACS Sustainable Chem. Eng.* **2016**, *4*, 6551-6561.

4. Hu, S.; Chen, X.; Torkelson, J.M. Biobased reprocessable polyhydroxyurethane networks: Full recovery of crosslink density with three concurrent dynamic chemistries. *ACS Sustainable Chem. Eng.* **2019**, *7*, 10025-10034.

5. Liu, X.; Yang, X.; Wang, S.; Wang, S.; Wang, Z.; Liu, S.; Xu, X.; Liu, H.; Song, Z. Fully bio-based polyhydroxyurethanes with a dynamic network from a terpene derivative and cyclic carbonate functional soybean oil. *ACS Sustainable Chem. Eng.* **2021**, *9*, 4175-4184.

6. Yang, X.; Ren, C.; Liu, X.; Sun, P.; Xu, X.; Liu, H.; Shen, M.; Shang, S.; Song, Z. Recyclable non-isocyanate polyurethanes containing a dynamic covalent network derived from epoxy soybean oil and CO2. *Mater. Chem. Front.* **2021**, *5*, 6160-6170.

7. Yang, X.; Wang, S.; Liu, X.; Huang, Z.; Huang, X.; Xu, X.; Liu, H.; Wang, D.; Shang, S. Preparation of non-isocyanate polyurethanes from epoxy soybean oil: Dual dynamic networks to realize self-healing and reprocessing under mild conditions. *Green Chem.* **2021**, *23*, 6349-6355.

8. Seychal, G.; Ocando, C.; Bonnaud, L.; De Winter, J.; Grignard, B.; Detrembleur, C.; Sardon, H.; Aramburu, N.; Raquez, J.-M. Emerging polyhydroxyurethanes as sustainable thermosets: A structure–property relationship. *ACS Appl. Polym. Mater.* **2023**, *5*, 5567-5581.

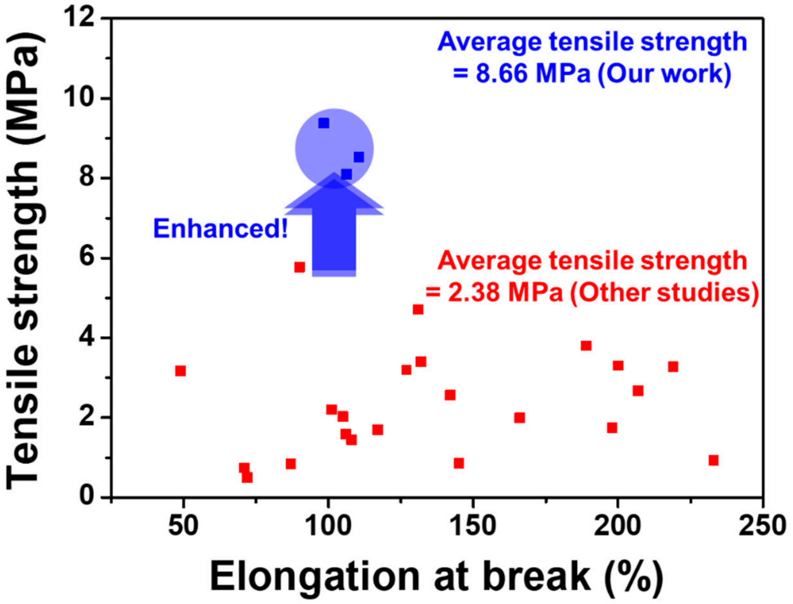

Figure S1. Tensile strength and elongation at break comparison of CSBO-based NIPU : Our work versus previous studies.

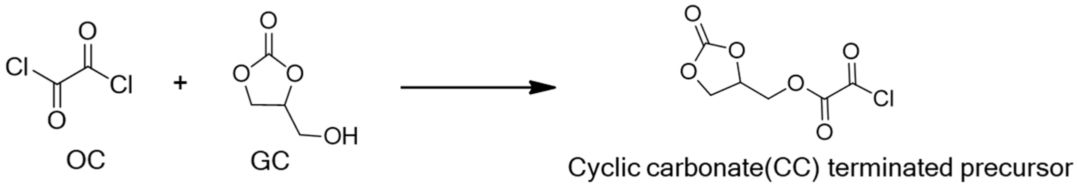

Figure S2. Schematic representation of the preparation of five-membered CC precursor.

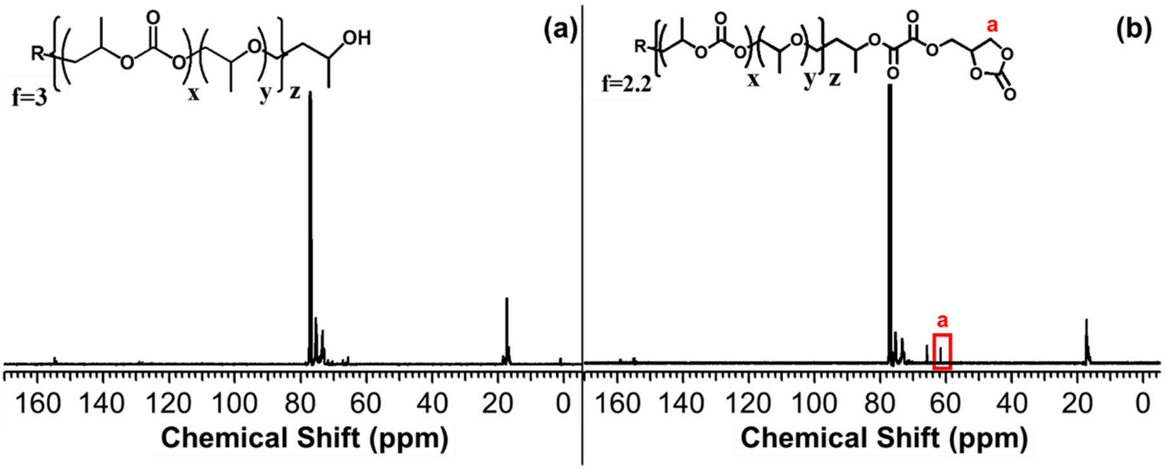

Figure S3. ^{13}C NMR spectra of PEC polyol (a) and RCC (b).

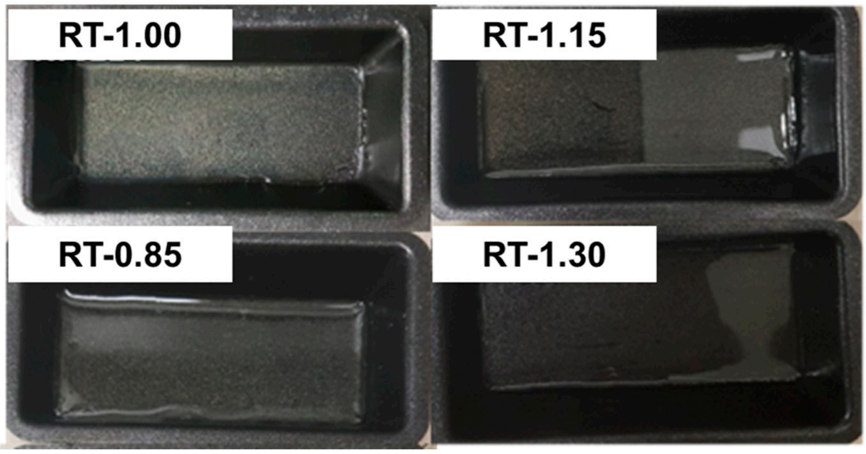

Figure S4. Visual appearances of RTs in iron mold.
